# The Hidden Habit of the Entomopathogenic Fungus *Beauveria bassiana*: First Demonstration of Vertical Plant Transmission

**DOI:** 10.1371/journal.pone.0089278

**Published:** 2014-02-14

**Authors:** Enrique Quesada-Moraga, Cristina López-Díaz, Blanca Beatriz Landa

**Affiliations:** 1 Department of Agricultural and Forest Sciences and Resources, Agricultural and Forestry Engineering School, University of Córdoba, Córdoba, Spain; 2 Institute for Sustainable Agriculture, Spanish National Research Council, Córdoba, Spain; Institute for Plant Protection (IPP), CNR, Italy

## Abstract

*Beauveria bassiana* strain 04/01-Tip, obtained from a larva of the opium poppy stem gall wasp *Iraella luteipes* (Hymenoptera; Cynipidae), endophytically colonizes opium poppy (*Papaver somniferum* L.) plants and protects them against this pest. The goal of this study was to monitor the dynamics of endophytic colonization of opium poppy by *B. bassiana* after the fungus was applied to the seed and to ascertain whether the fungus is transmitted vertically via seeds. Using a species-specific nested PCR protocol and DNA extracted from surface-sterilised leaf pieces or seeds of *B. bassiana-*inoculated opium poppy plants, the fungus was detected within the plant beginning at the growth stage of rosette building and them throughout the entire plant growth cycle (about 120–140 days after sowing). The fungus was also detected in seeds from 50% of the capsules sampled. Seeds that showed positive amplification for *B. bassiana* were planted in sterile soil and the endophyte was again detected in more than 42% of the plants sampled during all plant growth stages. *Beauveria bassiana* was transmitted to seeds in 25% of the plants from the second generation that formed a mature capsule. These results demonstrate for the first time the vertical transmission of an entomopathogenic fungus from endophytically colonised maternal plants. This information is crucial to better understand the ecological role of entomopathogenic fungi as plant endophytes and may allow development of a sustainable and cost effective strategy for *I. luteipes* management in *P. somniferum*.

## Introduction

The symbiosis between plants and entomopathogenic Ascomycetes such as *Beauveria bassiana* (Bals.) Vuill., *Isaria* sp., *Lecanicillium* spp. (Ascomycota: Hypocreales) etc., has become an important area of study in crop protection, agronomy and ecology over recent years [1]. This interest has been triggered not only because endophytic behaviour is a newly discovered aspect of the life style of arthropod pathogenic fungi, but also because entomopathogenic Ascomycetes symbioses can positively impact plant growth, resistance against invertebrate pests and fungal pathogens [1] [2] [3] [4].

We have described such an endophytic association between *B. bassiana* and opium poppy *Papaver somniferum* L., one of the oldest medicinal plants that is today the commercial source of important narcotic analgesics such as morphine [5] [6]. *Beauveria bassiana* strain EABb 04/01-Tip was originally isolated from naturally infected larvae of the stem gall wasp *Iraella luteipes* (Thompson) (Hymenoptera: Cynipidae) that were found dead within opium poppy stems. *Iraella luteipes* is a serious pest of opium poppy throughout most of the insect’s range of distribution [5]. Subsequently, it was shown that *B. bassiana* strain EABb 04/01-Tip can become an endophyte in the plant and systemically protects opium poppy against the stem gall wasp [5] [6]. We recently developed a two-step nested PCR protocol for specific identification and detection of *B. bassiana* in opium poppy tissues [7]; the assay is highly sensitive and detects as low as 10 fg of *B. bassiana*. This molecular tool was used to demonstrate that *B. bassiana* strain EABb 04/01-Tip, when applied to leaves as a conidial suspension, effectively colonized aerial tissues of opium poppy plants mainly through intercellular spaces and even leaf trichomes [7].

Until now, most studies of symbioses between plants and entomopathogenic Ascomycetes have focused on documenting the benefits of symbiosis to plant (crop) host fitness and tolerance to biotic factors, mainly pest and diseases, with very few studies focusing on the direct interaction between endophytic entomopathogenic Ascomycetes and their host plant [4]. Understanding the dynamics of fungal colonization of plant tissues and the mode of transmission of entomopathogenic Ascomycetes, if any, is important to identifying the incidence of an endophytic entomopathogenic Ascomycete in the host crop. It has been proposed that transmission of Clavicipitaceous endophytes, Class 1 endophytes [8], is primarily vertical, with maternal plants passing fungi on to offspring via seed infections [9]. Nonetheless, to our knowledge, no such information exists on the mechanism(s) of transmission of endophytic entomopathogenic Ascomycetes in a host.

In the present study, molecular tools were used to: (1) monitor the dynamics of colonization of *B. bassiana* strain EABb 04/01-Tip in opium poppy tissues during various stages of plant phenological development after applying the strain to the seed and; (2) determine whether *B. bassiana* strain EABb 04/01-Tip is transmitted to progeny from endophytically colonised maternal plants.

## Results and Discussion

**Figure 1:**
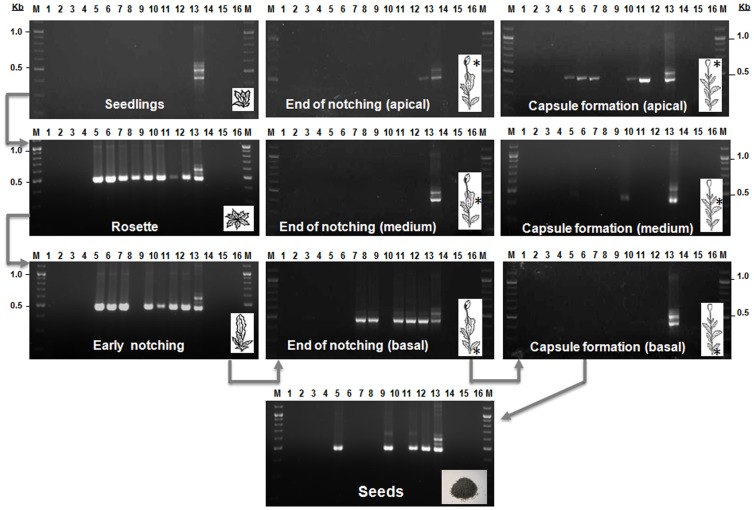
Monitoring of endophytic colonization by *Beauveria bassiana* strain EABb 04/01-Tip of opium poppy plants at different stages of growth. Surfaced sterilised seeds were treated with the endophyte and sown in sterile soil. DNA was extracted from surface sterilised pieces of plant sampled at different growth stages (seedling, rosette, early notching, end of notching, capsule formation, and seeds). M, ÓGene Ruler DNA ladder mix (Fermentas international Inc., Burlington, Ontario, Canada ); lanes: 1 to 4, Control plants (not inoculated); lanes 5 to 12, seed-dressed inoculated plants; lane 13, positive control (*B. bassiana* DNA); lane 14, negative control (water, 1^st^-PCR round); lane 15, negative control (water, 2^nd^-PCR round); lane 16 (empty lane). (*) Area sampled. Lane numbers for the different growth stages do not refer to the same plants, each plant was sampled only once.

Our first objective was to ascertain whether *B. bassiana* strain EABb 04/01-Tip can effectively establish as an endophyte and colonize different opium poppy tissues at various stages of plant growth after spores were applied as a seed treatment. Using our PCR protocol and DNA extracted from surface-sterilised leaf pieces of *B. bassiana* seed-inoculated opium poppy plants, we detected the endophyte in 100% (8/8 plants), 87.5% (7/8 plants), 62.5% (5/8 plants) and 62.5% (5/8 plants) during the growth stages of rosette, early notching, end of notching, and capsule formation, respectively (Figure 1). Interestingly, at the end of notching, *B. bassiana* was detected mainly at the basal part of the plant, whereas at capsule formation it was detected inside the leaves at the plant tip, indicating that the *B. bassiana* strain had adapted its growth strategy to reach the reproductive tissues, ensuring transmission to progeny from endophytically colonised maternal plants (Figure 1). The endophyte was detected in seeds from 50% (4/8 plants) of the capsules sampled (Figure 1). We ensured that the colonization of *B. bassiana* was indeed endophytic by analysing with the specific PCR assay aliquots of the washing water used for surface sterilisation of the leaves. In all samples of the washings *B. bassiana* inoculum was absent (*data not shown*). We also confirmed that the amplified products were identical to the expected ITS rDNA sequence of *B. bassiana* strain EABb 04/01-Tip (GenBank accession numbers DQ364698 and DQ 364699) by sequencing six PCR amplification products from randomly selected plants (Figure 1). In a previous study, similar or higher percentages of detection in leaves occurred shortly after the strain was sprayed onto the leaves (i.e., 2 to 5 days after inoculation); however, the ability to detect *B. bassiana* decreased in within a few days although fungal hyphae still remained present in the leaf tissues [7]. It is notable that in this study, the strain could be monitored within the plant from early stages of rosette (about 35 days) through the entire plant cycle (i.e., up to capsule drying, about 120–140 days). A long association (60 days) of a GFP-tagged strain of the entomopathogen *Metarhizium robertsii* and haricot beans has been reported, in which the fungus endophytically colonized cortical cells of roots; however the fungus did not move vertically throughout the plant [4].

**Figure 2:**
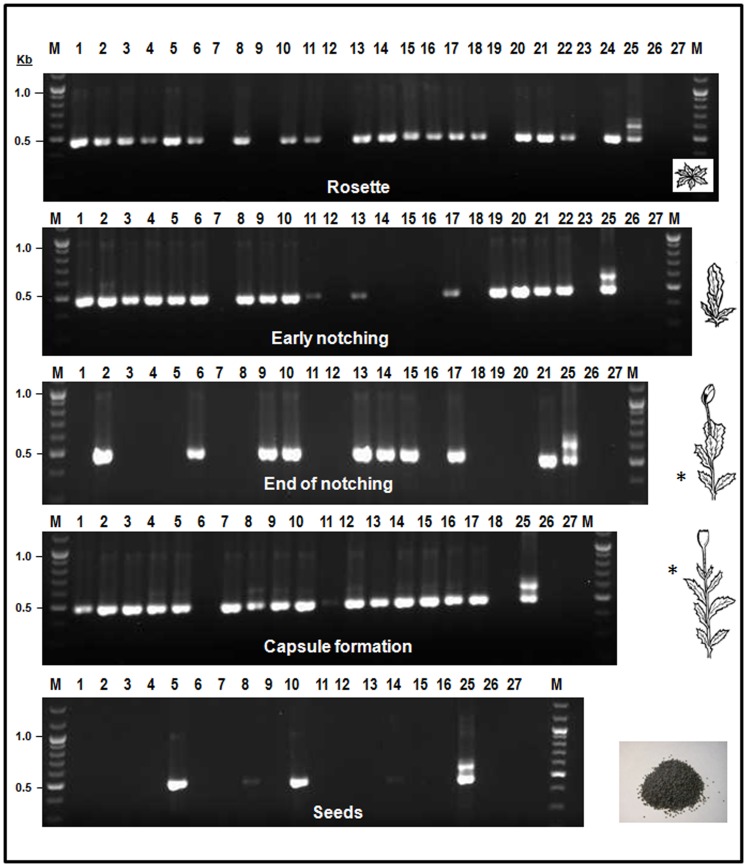
Monitoring of vertical transmission of *Beauveria bassiana* strain EABb 04/01-Tip. Seeds that showed a positive amplification for *B. bassiana* from Figure 1 were sown in a sterile soil. DNA was extracted from surface sterilised pieces of plant sampled at different stages of growth (rosette, early notching, end of notching, capsule formation, and seeds). M, ÓGene Ruler DNA ladder mix (Fermentas international Inc., Burlington, Ontario, Canada ); lanes: 1 to 24, plants grown from seed lots that showed positive amplification; lane 25, positive control (*B. bassiana* DNA); lane 26, negative control (water, 1st-PCR round); lane 27, negative control (water, 2nd-PCR round). (*) Area sampled. Lane numbers for the different growth stages do not necessarily refer to the same plant.

Our second objective was to determine whether *B. bassiana* strain EABb 04/01-Tip is transmitted to progeny from endophytically colonised maternal plants. For that purpose, seeds (approximately 20 seeds per capsule) obtained from three of the four capsules from the previous experiment that showed positive amplification for *B. bassiana* (Figure 1), were surface-sterilised before planting them into a sterile soil mixture. Of the surface-sterilised seeds, 24 germinated, 23 plants grew up to notching, and of those, 16 formed a mature capsule with seeds (Figure 2). We demonstrated vertical spread of the endophyte (as indicated by a positive PCR amplification) in 79.2% (19/24 plants), 69.6% (16/23 plants), 42.9% (9/21 plants) and 88.9% (16/18 plants) of plants from the growth stages rosette, early notching, end of notching, and capsule formation, respectively (Figure 2). More importantly, *B. bassiana* was transmitted to seeds in 25% (4 positive PCR samples) of 16 plants from the second generation that formed a mature capsule (Figure 2).

Most studies on the mode of transmission of fungal endophytes have focused on systemic fungal endophytes (Clavicipitaceae) of grasses [10]. These fungi can be transmitted either horizontally by sexual spores from infected individuals in the population or vertically from infected plants to offspring via seeds [10] [11]. Studies on vertically transmitted endophytes from seed are very scarce [12]. *Neotyphodium* endophytes and some *Epichloë* species (e.g. *E. festucae* Leuchtm., Schardl and M.R. Siegel, and *E. sylvatica* Leuchtm. and Schardl) are vertically transmitted to host progeny from infected seeds. Close to 100% of the seeds produced by an infected plant contained fungal mycelium near the embryo and in the aleurone layer [10], and these seeds gave rise to asymptomatic infected plants [10].

Results from our study have shown for the first time that *B. bassiana* EABb 04/01-Tip strain can be vertically transmitted through maternal plants. Although we have not elucidated yet which internal seed tissues are colonized by the fungus, nonetheless, to the best of our knowledge, no such information on this transmission mechanism in endophytic entomopathogens has been published to date. Additionally, the potential role of insect pests in disseminating endophytic fungi to their host plants has been recently highlighted [13]. In this context, the possible role of *I. luteipes* in disseminating the endophytic *B. bassiana* strain EABb 04/01-Tip in natural and cultivated populations of opium poppy and even in natural populations of *P. rhoeas* remains to be elucidated. Nevertheless, results from this study have provided key insights to formulate a sustainable and cost effective strategy for *I. luteipes* management in *P. somniferum*.

## Materials and Methods

### Fungal Isolate

The *B. bassiana* strain EABb 04/01-Tip isolated from a dead *Iraella luteipes* larva collected from a field in Carmona (Seville) and shown to behave as an endophytic strain [5] when inoculated into opium plant, was used throughout the study. This strain was deposited at the C.R.A.F. University of Cordoba Entomopathogenic Fungi Collection, Cordoba, Spain, and at the Spanish Collection of Culture Types (CECT), University of Valencia, with accession n°. CECT 20744. *B. bassiana* strain was routinely grown on slants of Malt Agar (MA; Biocult, Madrid, Spain) at 25°C in the dark and stored at 4°C.

### Plant Material

Seeds of commercial opium poppy cv. Nigrum provided by ALCALIBER S.A. (Carmona, Sevilla, Spain) were used throughout the study. Seeds were surface-sterilised in 1% NaOCl for 5 min, rinsed three times with sterile, ultrapure water, dried on sterile filter paper in a laminar flow hood for 30 min and treated with the *B. bassiana* strain. To ensure that the surface sterilisation was effective, some of the sterilised seeds were plated onto Sabouraud Dextrose Chloramphenicol Agar (SDCA; Biocult, Madrid, Spain) medium and incubated as described above. No microbial growth was observed after two weeks of incubation.

### DNA Extraction and Quantification

For positive PCR amplification controls DNA was extracted from actively growing cultures of the *B. bassiana* strain EABb 04/01-Tip grown onto a film of sterile cellophane layered over a plate of SDCA. Inoculated plates were incubated for 5 to 7 days at 25°C in the dark. Mycelium grown on the cellophane surface was removed by scraping the surface with a sterile scalpel, lyophilized, and stored at –20°C until used. Genomic DNA of the fungal strain was purified from 100 mg of lyophilized mycelium [7].

Genomic DNA of opium poppy seeds and leaves was extracted using the G-SpinTM IIp Plant Genomic DNA extraction kit (Intron Biotechnology, Korea) and the Fast Prep System Bio 101 (Qbiogene) as described in our previous studies [7] [14].

All DNA samples were quantified using the Quant-iT DNA Assay Kit Broad Range fluorometric assay (Molecular Probes Inc., Leiden, The Netherlands) and a Tecan Safire fluorospectrometer (Tecan Spain, Barcelona, Spain) [14]. Genomic DNA was diluted with sterile ultrapure water as appropriate.

### *B. bassiana* Specific Nested PCR Protocol

A two-step nested PCR protocol [7] using primers ITS1-F/ITS-4 and Bb.fw/Bb.rv, in the first and second round, respectively was used to detect endophytic colonization of the *B. bassiana* strain. PCR conditions for the first round were (final volume of 25 µl) 2.5 µl of 10× reaction buffer, 1.0 µM of each ITS-1F/ITS-4 primer, 50 µM of each dNTP, 2 units of FIREPol polymerase (Solis BioDyne, Tartu, Estonia), 1.5 mM MgCl_2_, and 1 µl of template DNA (20 ng of DNA). The cycling program included an initial denaturation step of 4 min at 95°C, followed by 40 cycles of 1 min denaturation at 94°C, 1 min annealing at 61°C, and 1 min extension at 72°C and a final 10 min extension step at 72°C followed by a 4°C soak. PCR conditions for the second round PCR were (final volume of 25 µl) 2.5 µl of 10× reaction buffer, 1.0 µM of each BB.fw/BB.rv primer, 50 µM of each dNTP, 1.5 units of DNA Polymerase, 1.5 mM MgCl2 and 1 µl of the template DNA of the first PCR product. The cycling program was similar to that of the first round PCR with 2 min at 95°C of initial denaturalization and 1 min annealing at 65°C. All PCR reactions included a positive control (*B. bassiana* DNA) and negative controls (no DNA) in the first and second reaction.

Amplification products were separated by electrophoresis in 1.5% agarose gels in 1× TAE buffer for 60 min at 80 V, stained with SafeView nucleic acid stain (NBS Biologicals Ltd, Cambridgeshire, UK) and visualized under UV light. The Gene-ruler™ DNA ladder mix (Fermentas, St Leon-Rot, Germany) was used for electrophoresis.

### Seed Treatment with the Endophytic *B. bassiana* Strain

To obtain a spore suspension, the *B. bassiana* strain EABb 04/01-Tip was grown on Petri plates on MA for 15 days at 25°C in the dark. Conidial suspensions were prepared by scraping conidia from Petri plates into an aqueous sterile solution of 0.002% Tween 80. The conidial suspensions were filtered through several layers of cheesecloth to remove mycelium mats, and sonicated for 10 minutes to homogenise the inoculum. Concentrations of viable conidia used for inoculation were determined using a haematocytometer and diluted in 0.01% Tween 80 to obtain a suspension of 1×10^8^ spores/ml. Viability of conidia was checked before preparation of suspensions by germination tests in liquid Czapek-Dox broth plus 1% (w/v) yeast extract medium. In all experiments, germination rates were higher than 90%.

Surfaced sterilized seeds were immersed in the conidial suspension for 30 minutes on a rotatory carrousel. Seeds were dried on sterile filter paper in a laminar flow hood for 30 min. Control seeds were treated with 0.01% Tween 80 only. The number of viable conidia of *B. bassiana* strain EABb 04/01-Tip on opium poppy seeds was estimated by placing three sets of 25 treated seeds in tubes with 10 ml of sterile distilled water, sonicating the suspension for 10 min, and vortexing for 1 min. Serial dilutions of the suspensions were plated onto SDCA. Plates were incubated for 10 days under the same conditions described before, andthe mean population density of *B. bassiana* was of 1.7±0.5×10^4^ cfu/seed.

Monitoring of endophytic opium poppy colonization and vertical transmission of *B. bassiana* strain EABb 04/01-Tip via nested PCR protocol.

*Beauveria bassiana* treated and untreated seeds were sown in a sterile (120°C, 45 min, 2 cycles) soil mixture (clay loam/peat, 2∶1, vol/vol) in 350 cm^3^ pots and incubated in a growth chamber (Sanyo MLR-350 H, Sanyo Electric Co., Ltd. Japan) at 23°C±1, 50/90% relative humidity (RH) and a 12-h photoperiod of fluorescent light at 360 µE·m^–2^·s^–1^. Water was provided daily as needed and sterile 50% Hoagland solution (Hoagland & Arnon, 1950) was added weekly to the pots. There were 50 or 25 replicated pots (one plant per pot) for the inoculated or control seeds, respectively.

Colonisation of plants by *B. bassiana* strain EABb 04/01-Tip was determined at different plant growth stages (GS) including seedling (GS1), rosette (GS2), early notching (GS3), end of notching (GS4), capsule formation (GS5) and from seeds formed in the capsules (GS6). At each sampling time, a series of eight inoculated plants or four control plants (not inoculated) were randomly selected. Each plant (pot) was sampled only once. Plants from GS1 were destructively sampled due to small size. For GS2 and GS3 the leaf base close to the stem was preferentially sampled from randomly selected leaves. For stages GS4 and GS5 three leaves from different parts of the plant were sampled including basal, medium and apical leaves. For GS6 a small amount of seeds was used for PCR detection to ensure enough viable germinating seeds for the next generation.

All leaves were surface-sterilised with 1% sodium hypochlorite for 2 min, rinsed twice in sterile distilled water, and dried on sterile filter paper. Pieces of leaves (each approximately 2 cm^2^) were cut with a sterile scalpel and immediately frozen at −20°C. Frozen samples were lyophilized and ground before DNA extraction as previously described [7]. *B. bassiana* treated seeds or seeds from second-generation plants were not surfaced sterilized and processed directly for DNA extraction as described above. DNA isolated from plant samples were processed using the two-step nested PCR protocol as described above. The universal primers ITS-5 and ITS-4 [15] were used as described above as an internal positive control for successful PCR amplification and to test for the presence of PCR inhibitors.

To demonstrate the vertical transmission of *B. bassiana* strain EABb 04/01-Tip, at least 20 surface sterilised seeds from each of three capsules (namely 5, 9 and 11) that showed a positive PCR amplification signal for *B. bassiana* were planted in pots containing sterile soil (one seed per pot), and incubated and grown as described above. Leaf and seed samples from the same plant from growth stages GS2, GS3, GS4, GS5 and GS6 were sampled and processed as described above for PCR detection of the *B. bassiana* strain using the two-step nested specific-PCR protocol [7]. Opium poppy seeds showed 50% germination with 28 plants reaching the rosette stage. Seedlings (GS1) were not sampled because at this growth stage sampling would have meant destroying the plant; therefore, making impossible the inspection of further growth stages and the demonstration of vertical transmission.

### Sequencing of the ITS Region of *B. bassiana* Strain EABb 04/01-Tip

To demonstrate that the amplified product from second-round PCR corresponded to the *B. bassiana* strain EABb 04/01-Tip, six PCR products were randomly chosen from second-round PCR and purified using a gel extraction kit (FavorPrepTM Gel/PCR Purification, Favorgen, Taiwan), quantified with the Quant-iT DNA Assay Kit Broad Range fluorometric assay as described before, and used for direct DNA sequencing with primers Bb.fw/Bb.rv in both directionsusing a terminator cycle sequencing ready reaction kit (BigDye, Perkin-Elmer Applied Biosystems, Madrid, Spain) according to the manufacturer’s instructions at the STABVIDA sequencing facilities (Caparica, Portugal).
